# Development of an Open-Heart Intraoperative Risk Scoring Model for Predicting a Prolonged Intensive Care Unit Stay

**DOI:** 10.1155/2014/158051

**Published:** 2014-04-10

**Authors:** Sirirat Tribuddharat, Thepakorn Sathitkarnmanee, Kriangsak Ngamsangsirisup, Somrat Charuluxananan, Cameron P. Hurst, Suparit Silarat, Ganjana Lertmemongkolchai

**Affiliations:** ^1^Department of Anesthesiology, Faculty of Medicine, Khon Kaen University, Khon Kaen 40002, Thailand; ^2^Department of Anesthesiology, Faculty of Medicine, Chulalongkorn University, Bangkok 10330, Thailand; ^3^Clinical Epidemiology Unit (CEU), Faculty of Medicine, Khon Kaen University, Khon Kaen 40002, Thailand; ^4^Department of Clinical Immunology, Faculty of Associated Medical Sciences, Khon Kaen University, Khon Kaen 40002, Thailand

## Abstract

*Background*. Based on a pilot study with 34 patients, applying the modified sequential organ failure assessment (SOFA) score intraoperatively could predict a prolonged ICU stay, albeit with only 4 risk factors. Our objective was to develop a practicable intraoperative model for predicting prolonged ICU stay which included more relevant risk factors. *Methods*. An extensive literature review identified 6 other intraoperative risk factors affecting prolonged ICU stay. Another 168 patients were then recruited for whom all 10 risk factors were extracted and analyzed by logistic regression to form the new prognostic model. *Results*. The multivariate logistic regression analysis retained only 6 significant risk factors in the model: age ≥ 60 years, PaO_2_/FiO_2_ ratio ≤ 200 mmHg, platelet count ≤ 120,000/mm^3^, requirement for inotrope/vasopressor ≥ 2 drugs, serum potassium ≤ 3.2 mEq/L, and atrial fibrillation grading ≥2. This model was then simplified into the Open-Heart Intraoperative Risk (OHIR) score, comprising the same 6 risk factors for a total score of 7—a score of ≥3 indicating a likely prolonged ICU stay (AUC for ROC of 0.746). *Conclusions*. We developed a new, easy to calculate OHIR scoring system for predicting prolonged ICU stay as early as 3 hours after CPB. It comprises 6 risk factors, 5 of which can be manipulated intraoperatively.

## 1. Introduction 

Cardiac surgery with cardiopulmonary bypass (CPB) is associated with immune responses harmful to the patient, often called “post-bypass syndrome” [1, 2]. It may contribute to life-threatening perioperative complications and even multiorgan dysfunction syndrome (MODS) or multiorgan failure (MOF), which causes considerable clinical morbidity, mortality, and eventually a prolonged stay in the intensive care unit (ICU)/hospital [3, 4]. Early evaluation of the severity of the intraoperative immunoinflammatory response, with a proper predictive model, is helpful for (a) recognizing the possible increase in risk, (b) planning proper management, (c) reducing postoperative complications, (d) improving outcomes, and (e) decreasing the length of stay in intensive care (ICU) and hospital.

There are several predictive models for prolonged ICU stay, but all are used preoperatively for proper resource management [5]. What is lacking, therefore, is a predictive model for intraoperative use, which identifies correctable risk factors that can be addressed for improving patient care and prognosis.

Prognostic scoring systems for assessing the severity of inflammation and predicting the mortality rate and length of ICU stay include (a) the acute physiology and chronic health evaluation (APACHE) II; (b) the sequential organ failure assessment (SOFA); and (c) the multiorgan dysfunction (MOD) scores [6]. SOFA is the simplest to calculate at bedside and may be used to predict prolonged stay in ICU after cardiac surgery. We conducted a pilot study among 34 patients undergoing cardiac surgery with CPB to assess the ability of a modified SOFA score for predicting prolonged ICU stay. We, however, excluded the Glasgow coma score because such patients were unconscious from general anesthesia. The modified SOFA 3 hours after CPB initiation predicted a prolonged ICU stay but was not suitable for intraoperative assessment because it comprises only 4 known relevant risk factors, namely, PaO_2_/FiO_2_ (P/F) ratio, inotropic/vasopressor drugs used, platelet count, and creatinine level. A new intraoperative prognostic model with more relevant correctable risk factors is needed.

Our objective was to develop a new, simple, prognostic model to be used intraoperatively to predict prolonged ICU stay and as a tool for improving patient care in cardiac surgery.

## 2. Methods

This was a prospective, observational, exploratory, analytical study. The experimental protocol was approved by the Human Research Ethics Committee, Khon Kaen University (HE531033 and HE541122), and registered at ClinicalTrials.gov (NCT01353157 and NCT01559870). The pilot study was conducted between February and June 2010 and the model development between July and December 2011 at Queen Sirikit Heart Center of the Northeast, Khon Kaen University, Thailand. All of the patients gave written, informed consent before recruitment.

An extensive literature review revealed another 6 relevant risk factors influencing prolonged ICU stay [7–18], namely, age, New York Heart Association (NYHA) classification, ejection fraction, hematocrit level, serum potassium level, and atrial fibrillation grading. According to Tabachnick and Fidell [19], the required sample size (*N*) for a logistic regression of a full model should ideally be 50 + 10(*k*): for us this meant 168 patients based on 10 relevant clinical risk factors (*k*) and a dropout margin of 10%.

We included any patient, between 18 and 75 years of age, with acquired heart disease (including coronary artery disease or valvular heart disease), scheduled for elective cardiac surgery with CPB. We excluded any patient with congenital heart disease and those requiring emergency or redo surgery. Standard anesthetic and surgical techniques were employed for the open-heart surgery with CPB. The anesthesiologists and surgeons were not apprised of the risk factors being studied. A transfusion was indicated in case of (a) hemoglobin level < 8 g/dL, (b) platelet number < 50,000/mm^3^, or (c) clinical coagulopathy. The target of fluid management was to maintain a central venous pressure of 8 to 12 mmHg or pulmonary arterial pressure of 12 to 15 mmHg by infusion of crystalloid and colloids. As per protocol, catecholamine infusions were used to support hemodynamic stability, and the sequence of their use was dobutamine then epinephrine or norepinephrine.

After surgery, all of the patients were transferred to the ICU and managed as follows. Before extubation, ventilated patients must (a) be awake and (b) have satisfactory oxygenation and ventilation (i.e., on FiO_2_ ≤ 40% with PaO_2_ > 60 mmHg, PaCO_2_ > 30 and < 50 mmHg, and pH > 7.30, or SpO_2_ > 92%) and (c) be hemodynamically stable. An attending surgeon discharged each patient from the ICU to the cardiovascular ward according to the following criteria: the patient must (a) be alert and cooperative, (b) have a respiratory rate < 25/minute without assistance from mechanical ventilation, (c) have a PaO_2_ > 80 mmHg and a PaCO_2_ < 45 mmHg, (d) be hemodynamically stable, and (e) have adequate analgesia.

All 10 risk factors were evaluated 3 hours after CPB. Perioperative data were collected, including clinical outcomes and complications. Prolonged ICU stay was defined as an ICU stay of longer than the 42-hour median time in our hospital. All data were extracted for analyses by 2 physicians. The data were checked and any entry errors corrected before the analyses.

### 2.1. Statistics

The discrimination ability of each predictor was evaluated using the area under the receiver operating characteristic curve (AUC for ROC), for which values ≥ 0.600 were considered clinically useful. After determination of the best cut-off points (i.e., by choosing the points with maximum area of the ROC), the continuous values for all of the risk factors were transformed into dichotomous values. Each risk factor was evaluated for the crude odds ratio using the results of the univariate analysis. All of the risk factors with a *P* < 0.20 were included in the multivariate logistic regression analysis. Calibration and validation of the model were accomplished using the Hosmer-Lemeshow goodness-of-fit statistic and external validation. The *α* was set at 0.05. All analyses were performed using GraphPad Prism 5 (GraphPad software, La Jolla, CA, USA) and SPSS version 16.0 for Windows (SPSS, Chicago, IL, USA).

## 3. Results 

The demographic and clinical data of 168 patients are presented in Table 1.  Table 2 presents the AUC for ROC, crude odds ratios, sensitivity, and specificity for all of the risk factors for predicting prolonged ICU stay. Based on the multivariate logistic regression analysis, only 6 risk factors should be retained in the equation. The regression coefficients and adjusted odds ratios are presented in Table 3. The logistic regression model for predicting the probability of a prolonged ICU stay (P) is

(1) P=1×(1+EXP(−(−2.576+0.974∗  “Age≥60  years”+0.857∗  “P/F  ratio≤200 mmHg”   +0.847∗  “Platelet  count≤120×103/mm3”    +1.593∗×“Inotrope/vasopressor  requirement≥2  drugs”       +0.910∗  “Serum  potassium≤3.2 mEq/L”     +0.934∗  “Atrial  fibrillation  grading≥2”)))−1,

where P = probability of a prolonged ICU stay; the values of each risk factor are assigned as follows:

 age ≥ 60 years (yes = 1; no = 0);
 P/F ratio ≤ 200 mmHg (yes = 1; no = 0);
 platelet count ≤ 120 × 10^3^/mm^3^ (yes = 1; no = 0);
 inotrope/vasopressor requirement ≥ 2 drugs (yes = 1; no = 0);
 serum potassium ≤ 3.2 mEq/L (yes = 1; no = 0);
 atrial fibrillation grading ≥ 2 (yes = 1; no = 0).

The AUC for ROC of this model was 0.818 (95% CI: 0.753–0.883) and, according to the Hosmer and Lemeshow goodness-of-fit test, the model fit the data (*P* = 0.242). Since this model was complex and difficult to calculate, we simplified it into a new Open-Heart Intraoperative Risk (OHIR) score by approximating the adjusted odds ratio around 2.5 as an OHIR score of 1 (Table 4).

**Figure 1:**
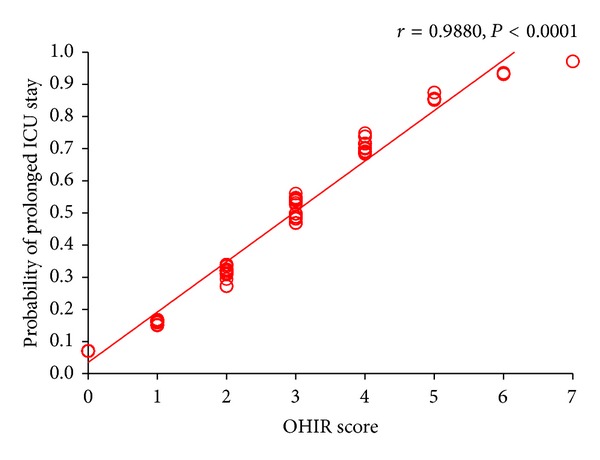
Correlation of Open-Heart Intraoperative Risk (OHIR) score and probability of a prolonged ICU stay, derived from the multivariate logistic regression model, presented as the Pearson correlation.

The new scoring model includes all 6 risk factors and has a total score of 7. An OHIR score of ≥3 indicates a prolonged ICU stay (Table 4). The sensitivity, specificity, positive and negative predictive value, overall accuracy, positive likelihood, and AUC for ROC of this model are presented in Table 5. The correlation of the logistic regression and OHIR score models was 0.988 (*P* < 0.0001, 95% CI: 0.9837–0.9911) (Figure 1).

Both the logistic regression and OHIR models were externally validated in the 34 patients of the pilot study. The respective AUC for ROC of both models in this validating group was 0.901 (95% CI: 0.789–1.014) and 0.915 (95% CI: 0.807–1.023). The respective sensitivity and specificity of the OHIR score ≥ 3 for predicting prolonged ICU stay were 81.2% (95% CI: 53.7%–95.0%) and 94.4% (95% CI: 70.6%–99.7%).

In order to confirm the validity of applying the OHIR score on our patients, we computed the mean ICU stay for each OHIR score for all of the patients (Table 6). The mean ICU stay increased with the OHIR score. Importantly, there was a significant gap between an OHIR score of 2 and 3 across our cut-off point of 42 hours.

During this study, there were neither deaths nor any serious complications, for example, coma, stroke, heart failure, acute renal failure, or respiratory failure.

## 4. Discussion

We developed the intraoperative predictive model (OHIR score) for a prolonged ICU stay after cardiac surgery with CPB. We first validated the modified SOFA score for its predictive capability in a pilot study. There are several prognostic scoring models being used in ICU to assess the risks of systemic inflammatory response, sepsis and severe sepsis, and MODS/MOF of critically ill patients (e.g., APACHE, SOFA, and MOD) [20–23]. These have, however, never been used to evaluate risk of, or predict, a prolonged ICU stay among patients undergoing cardiac surgery with CPB. If these scores can assess the severity of inflammation of patients in the ICU, they may be used in patients undergoing cardiac surgery to predict a prolonged ICU stay.

We chose the SOFA score because (a) it includes risk factors relevant to surgery and (b) it is practicable at bedside. We modified it by omitting the Glasgow coma score because the patients were unconscious under general anesthesia. The modified SOFA scores 3 hours after CPB insertion yielded the best prediction of a prolonged ICU stay. We chose all variables relevant at this juncture to develop the new model because this juncture is considered optimal for assessing prognosis since it is sufficiently early for the attending anesthesiologists to ameliorate the correctable risk factors so as to improve postoperative outcomes.

The modified SOFA score can accurately predict a prolonged ICU stay (with an AUC for ROC of 0.849). The Parsonnet and EuroSCORE, as well as the modified SOFA score, were initially developed to predict mortality but they also have the scope for predicting prolonged ICU stay [5, 24–26]. The modified SOFA score is inappropriate for intraoperative use as it consists of too few intraoperative risk factors (only 4), but it seeded development of this new model.

The definition of a prolonged ICU stay varies among studies from >24 hours to >3 days [5]. For inferential purposes, we used statistic-based criteria, that is, length of stay in ICU longer than the median, which was 42 hours in our hospital.

Our multivariate logistic regression model consisted of 6 risk factors. Although the original model had both high discrimination and precision, the formula was complicated and difficult to calculate. We, therefore, modified it into a simple scoring model and called it the OHIR score, which contains the same risk factors but with a simplified formula, easy to calculate at bedside. Both models provided excellent linear correlation indicating that they could substitute each other and both showed very good discrimination vis-à-vis the external validation test.

This is the first model for intraoperative use, to predict the prognosis of the patients undergoing cardiac surgery. From the model, all except one risk factor (i.e., age ≥ 60 years) are manageable, meaning that if these factors are improved in the patient, the OHIR score can be reduced and the probability of a prolonged ICU stay may be decreased.

Many predictive models have been developed for a prolonged ICU stay—for example, the Parsonnet, EuroSCORE, Tuman, TU, Pitkanen, Hujdkes, Christakis, Wong, Ivanov, Janssen, Abrahamyam, and Ghotkar models [5, 24, 25]. These are, however, for different populations with differences in definitions of outcomes and predictors. All of them, moreover, are preoperative evaluation tools, and none can be used as an intraoperative tool, unlike our model, which was developed by expanding the scope of the modified SOFA score. Our OHIR model consists of 6 risk factors, 5 of which are manipulatable. We suggest that it may be a useful intraoperative tool for (a) assessing prognosis, (b) providing the anesthesiologist with enough time to correct the risk factors, thereby (c) improving both anesthetic care and patient outcomes, and (d) lowering the costs of patient care.

### 4.1. Limitations

Although we planned to treat and discharge the patients according to the mentioned protocols, the time when the patients were transferred into or out of ICU may depend greatly on when the surgery was complete, availability of bed in ordinary ward, and the decision made in day or night shift. Although our sample size was small compared with other retrospective development models, this was a prospective study with an appropriate sample size calculation: our study, thus, demonstrates high discrimination and good precision. Despite the external validation of both the logistic regression and OHIR models in an independent group of patients, all of the patients remained in the same hospital. Further validation of the OHIR score in other institutions is, therefore, recommended.

## 5. Conclusions

We developed a simple intraoperative OHIR score for predicting prolonged ICU stay after cardiac surgery, starting with validation of the modified SOFA score. We used the risk factors from the modified SOFA score, plus those from an extensive literature review, to construct a predictive model using logistic regression analysis. The logistic regression model had good calibration and discrimination. This model was further simplified into the OHIR score. Both models had excellent correlation and were externally validated in the pilot study patients with good discrimination. The OHIR score is relatively simple and easy to calculate at bedside. It may be used as an intraoperative tool for improving the quality of anesthetic care.

## Figures and Tables

**Table 1:** Demographic and clinical data.

Characteristics	Nonprolonged ICU (*n* = 102)	Prolonged ICU (*n* = 66)	*P* value
Age (years)	54.5 ± 11.2	60.5 ± 12.3	0.002
Gender			
Male	61 (59.8)	54 (81.8)	0.004
Female	41 (40.2)	12 (18.2)	
Type of operation			
CABG	49 (48.0)	36 (54.6)	0.433
Valve surgery	49 (48.0)	22 (33.3)	0.078
CABG + valve surgery	4 (4.0)	8 (12.1)	0.023
NYHA class			
I-II	83 (81.4)	42 (63.6)	0.012
III-IV	19 (18.6)	24 (36.4)	
Ejection fraction (%)	59.2 ± 14.0	53.0 ± 16.5	0.012
<40	12 (11.8)	16 (24.2)	0.055
≥40	90 (88.2)	50 (75.8)	
Preoperative variables:			
Hypertension	33 (32.4)	32 (48.5)	0.051
Diabetes mellitus	27 (26.5)	22 (33.3)	0.386
Myocardial infarction	16 (15.7)	15 (22.7)	0.309
Dyslipidemia	13 (12.8)	16 (24.2)	0.062
Atrial fibrillation	20 (19.6)	12 (18.1)	0.844
Congestive heart failure	7 (6.7)	11 (16.7)	0.071
Kidney impairment/disease	4 (3.9)	7 (10.6)	0.155
Creatinine value (mg/dL)	0.95 ± 0.3	1.11 ± 0.5	0.025
CPB time (minutes)	107.9 ± 37.9	126.6 ± 36.9	0.002
Aortic cross-clamping time (minutes)	78.1 ± 29.8	89.4 ± 27.2	0.014
Mechanical ventilation (hours)	10.9 ± 7.5	31.8 ± 41.5	<0.001
Endotracheal tube retaining (hours)	11.7 ± 8.1	33.4 ± 41.5	<0.001
ICU stay (hours)	25.7 ± 9.3	88.8 ± 51.6	<0.001
Hospital stay (days)	13.6 ± 5.3	18.5 ± 9.9	<0.001

NYHA: New York Heart Association; CABG: coronary artery bypass graft surgery; MVR: mitral valve replacement; MV: mitral valve; AVR: aortic valve replacement; TV: tricuspid valve; CPB: cardiopulmonary bypass; ICU: intensive care unit. Values are mean ± SD or *n* (%).

**Table 2:** Univariate risk factors of a prolonged length of ICU stay.

Variable outcomes	AUC for ROC	95% CI	Crude OR	95% CI	Sensitivity (%)	Specificity (%)
Age (years)						
<60			1.0			
≥60	0.629	0.543–0.716	2.881**	1.518–5.467	62.1	63.7
NYHA classification						
I-II			1.0			
III-IV	0.589	0.499–0.678	2.496*	1.231–5.063	36.4	81.4
Ejection fraction (%)						
>40			1.0			
≤40	0.562	0.472–0.653	2.400*	1.052–5.474	42.4	79.4
Hematocrit (%)						
>33			1.0			
≤33	0.570	0.483–0.657	3.355*	1.202–9.366	90.9	19.6
PaO_2_/FiO_2_ ratio (mmHg)						
>200			1.0			
≤200	0.607	0.517–0.696	2.838**	1.420–5.674	40.9	80.4
Platelet count (/mm^3^)						
>120,000			1.0			
≤120,000	0.607	0.517–0.696	2.838**	1.420–5.674	40.9	80.4
Inotropic drugs (items)						
1			1.0			
≥2	0.672	0.586–0.758	4.932***	2.463–9.876	53.0	81.4
Creatinine (mg%)						
<1.2			1.0			
≥1.2	0.581	0.491–0.671	2.508*	1.192–5.277	31.8	84.3
Potassium level (mEq/L)						
>3.2			1.0			
≤3.2	0.578	0.487–0.668	2.812*	1.251–6.323	27.3	88.2
Atrial fibrillation grading^#^						
<2			1.0			
≥2	0.634	0.547–0.721	3.020**	1.588–5.742	59.1	67.6

ICU: intensive care unit; AUC for ROC: area under receiver operating characteristic curve; CI: confidence interval; OR: odds ratio; NYHA: New York Heart Association; PaO_2_: partial pressure of arterial oxygen; FiO_2_: fraction of inspired oxygen in a gas mixture.

^#^Atrial fibrillation (AF) grading: 0: no AF; 1: AF without tachyarrhythmia and needs no treatment; 2: AF with tachyarrhythmia and needs antiarrhythmic therapy; 3: AF with tachyarrhythmia and hemodynamic instability and needs antiarrhythmic therapy.

*P < 0.05, **P < 0.01, ***P < 0.001.

**Table 3:** Risk factors associated with a prolonged length of ICU stay from multivariate logistic regression analysis and the OHIR score.

Variable outcomes	Regression coefficient	Adjusted OR	95% CI	*P* value
Age (≥60 years)	0.974	2.648	1.217–5.762	0.014
P/F ratio (≤200 mmHg)	0.857	2.357	1.048–5.301	0.038
Platelet count (≤120,000/mm^3^)	0.847	2.333	1.011–5.383	0.047
Inotropic drugs (≥2 items)	1.593	4.920	2.212–10.943	<0.001
Potassium level (≤3.2 mEq/L)	0.910	2.485	0.965–6.394	0.059
Atrial fibrillation (grading ≥2)	0.934	2.545	1.196–5.418	0.015
Constant	−2.576	0.076	—	<0.001

ICU: intensive care unit; OHIR: Open-Heart Intraoperative Risk; OR: odds ratio; CI: confidence intervals.

**Table 4:** The OHIR score model for predicting a prolonged ICU stay.

Risk factors	Score	
	Present	Absent
Age (≥60 years)	1	0
P/F ratio (≤200 mmHg)	1	0
Platelet count (≤120,000/mm^3^)	1	0
Inotrope/vasopressor requirement (≥2 drugs)	2	0
Serum potassium (≤3.2 mEq/L)	1	0
Atrial fibrillation (grading ≥2)	1	0

OHIR score: Open-Heart Intraoperative Risk score; total score = 7; score ≥3 suggests a prolonged stay in intensive care unit.

**Table 5:** The sensitivity, specificity, positive predictive value, and negative predictive value of OHIR score at cut-off point ≥3 in this study.

OHIR score	Value (%)	95% CI
Sensitivity	72.3	60.2–82.6
Specificity	76.5	66.8–84.1
Positive predictive value	66.7	54.5–77.1
Negative predictive value	81.2	71.7–88.2
Accuracy	75	—
Likelihood ratio	3.09	—
AUC for ROC of OHIR	0.746	0.668–0.824

OHIR: Open-Heart Intraoperative Risk; CI: confidence intervals; AUC for ROC: the area under the receiver operating characteristic curve.

**Table 6:** Mean ICU stay for each OHIR score for the patients in this study.

OHIR score	Number of patients	Mean (hours)	SD
0	28	28.62	17.37
1	31	29.69	15.82
2	37	38.50	22.88
3	34	62.62	48.52
4	23	71.37	61.07
5	9	128.22	68.71
6	4	51.75	21.87
7	2	102.50	17.68

ICU: intensive care unit; OHIR: Open-Heart Intraoperative Risk; SD: standard deviation.
